# Supplementary material for: The Effects of Ultrasonic and Gamma Irradiation on the Flavor of Potato Wines Investigated by Sensory Omics
Source: Foods. 2023 Jul 25;12(15):2821. doi: 10.3390/foods12152821 (PMC10417215; doi:10.3390/foods12152821)
Supplement: Supplementary file 1 [file foods-12-02821-s001.zip › Figure S1.pdf]

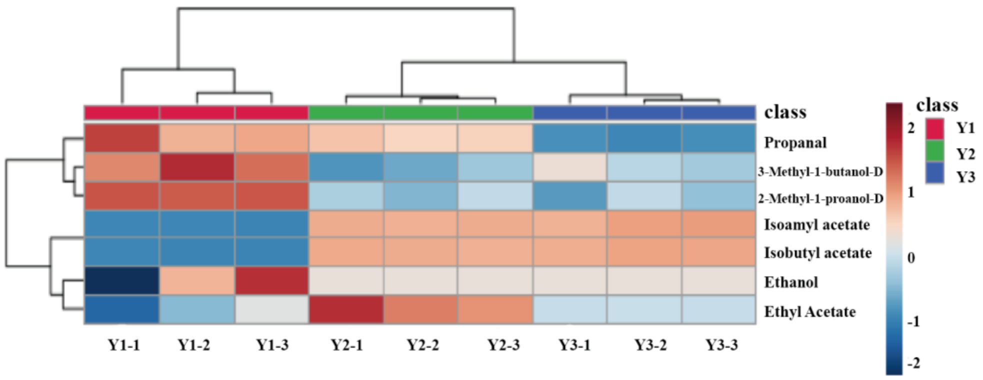

Figure S1. Cluster heat maps of different compounds (VIP > 1). Y1: newly produced potato wine; Y2: ultrasonic treated potato wine; Y3: gamma irradiated potato wine.
