# Supplementary material for: The Effects of Ultrasonic and Gamma Irradiation on the Flavor of Potato Wines Investigated by Sensory Omics
Source: Foods. 2023 Jul 25;12(15):2821. doi: 10.3390/foods12152821 (PMC10417215; doi:10.3390/foods12152821)
Supplement: Supplementary file 1 [file foods-12-02821-s001.zip › Table S1.pdf]

Table S1. E-nose sensors and their main application in PEN3

Sensor name	Performance description	Representative material species
W1C	Sensitive to aromatic constituents, benzene	Aromatic
W5S	Sensitive to nitrogen oxides	Broad range
W3C	Sensitive to aroma, ammonia	Aromatic compounds
W6S	Mainly selective for hydrides	Hydrogen
W5C	Short-chain alkane aromatic components	Arom-aliph
W1S	Sensitive to methyl	Broad-methane
W1W	Sensitive to sulfides	Sulphur-organic
W2S	Sensitive to alcohols, aldehydes and ketones	Broad-alcohol
W2W	Sensitive to aromatic ingredients and organic sulfides	Sulph-chlor
W3S	Sensitive to long-chain alkanes	Methane-aliph
