# Supplementary material for: The Effects of Ultrasonic and Gamma Irradiation on the Flavor of Potato Wines Investigated by Sensory Omics
Source: Foods. 2023 Jul 25;12(15):2821. doi: 10.3390/foods12152821 (PMC10417215; doi:10.3390/foods12152821)
Supplement: Supplementary file 1 [file foods-12-02821-s001.zip › Table S2.pdf]

Table S2. Aroma sensory evaluation criteria

aroma attribute	definition	reference
fruity	the smell of ripe fruit	20 mg /L Ethyl caproate in aqueous solution
alcohols	the aroma of alcohol components in Baijiu	20 mg /L isoamyl alcohol, ethanol 40% alcohol aqueous solution
cooked potato	the aroma of boiled potatoes	20mg/L 3-methylthiopropanal aqueous solution
sweet	the aroma of Baijiu that resembles sweetness	5μL honey dissolved in 50 mL water
fen-flavor	the aroma of Ethyl acetate as the main component in Baijiu	aqueous solution of 20mg/L koji
daqu aroma	Baijiu presents the aroma of the used koji and Daqu	aqueous solution of 120 mg /L ethyl acetate and 6 mg /L ethyl lactate
color	colorless or yellowish, clear and bright	ND
tasted	the Baijiu body is soft and harmonious, sweet and clean, with a long finish	ND
style	the style is typical of distilled wine and potato wine	ND
preference	the degree to which the reviewer likes liquor in general	ND
purity	no suspension, no precipitation	ND

ND: no reference to the sample.
