# Supplementary material for: The Effects of Ultrasonic and Gamma Irradiation on the Flavor of Potato Wines Investigated by Sensory Omics
Source: Foods. 2023 Jul 25;12(15):2821. doi: 10.3390/foods12152821 (PMC10417215; doi:10.3390/foods12152821)
Supplement: Supplementary file 1 [file foods-12-02821-s001.zip › Table S3.pdf]

Table S3. Volatile compounds identified in different potato wines by GC-MS.

no	Compound	Formula	RI	Relative content (%)		
				Y1	Y2	Y3
1	Ethyl alcohol	C_2H_6O	4.254	89.37	89.33	92.45
2	N-propanol	C_3H_8O	4.956	0.38	0.37	0.41
3	Isobutyl alcohol	$C_4H_{10}O$	5.950	1.11	0.95	0.74
4	(R)-(-)-2-butanol	$C_4H_{10}O$	5.525	0.69	0.78	0.69
5	Isoamyl alcohol	$C_5H_{12}O$	8.114	4.16	3.37	2.64
6	Ethyl acetate	$C_4H_8O_2$	5.688	1.72	1.90	1.2
7	Ethyl octanoate	$C_{10}H_{20}O_2$	14.965	0.35	0.69	0.32
8	Ethyl decanoate	$C_{12}H_{24}O_2$	16.837	1.73	1.63	0.94
9	Ethyl propionate	$C_5H_{10}O_2$	7.568	ND	0.22	0.14
10	1,1-Diethoxy-ethane	$C_6H_{14}O_2$	7.942	0.33	0.44	0.33
11	1,2-Dimethyl-3-ethylbenzene	$C_{10}H_{14}$	14.696	0.08	ND	ND
12	4-Isopropyltoluene	$C_{10}H_{14}$	14.700	ND	0.11	ND
13	1,2,4,5-Tetramethylbenzene	$C_{10}H_{14}$	14.321	ND	ND	0.04
14	1,2,3,4-Tetramethylbenzene	$C_{10}H_{14}$	14.319	ND	0.11	0.04

ND: Not detected in sample. Y1: newly produced potato wine; Y2: ultrasonic treated potato wine; Y3: Gamma irradiated potato wine.
