# Supplementary material for: The Effects of Ultrasonic and Gamma Irradiation on the Flavor of Potato Wines Investigated by Sensory Omics
Source: Foods. 2023 Jul 25;12(15):2821. doi: 10.3390/foods12152821 (PMC10417215; doi:10.3390/foods12152821)
Supplement: Supplementary file 1 [file foods-12-02821-s001.zip › Table S4.pdf]

Table S4. Volatile compounds identified in different potato wines by GC-IMS.

Count	Compound	RI	Rt [sec]	Dt [RIPrel]	Intensity (a.u.)		
					Y1	Y2	Y3
V1	Propanoic acid	1632.1	1692.95	1.11	236.52±42.51[b]	336.97±46.04[a]	417.44±34.14[a]
V2	Methional	1493.2	1252.46	1.09	679.24±59.42[a]	365.71±58.24[b]	283.67±2.43[b]
V3	Acetic acid	1499.0	1268.34	1.16	877.56±51.99[c]	1066.40±104.81[b]	1241.74±24.13[a]
V4	Ethyl octanoate	1448.0	1135.39	1.48	647.00±17.06[c]	1022.67±21.10[b]	1095.63±32.27[a]
V5	Ethyl lactate	1356.6	931.02	1.53	915.58±74.92[b]	1160.92±110.32[a]	1170.22±107.50[a]
V6	cis-2-Penten-1-ol	1299.7	823.01	0.956	214.23±2.86[a]	207.39±2.63[a]	180.05±18.88[b]
V7	Terpinolene	1295.3	815.21	1.21	81.22±13.09[a]	74.06±5.01[a]	89.15±1.67[a]
V8	p-Cymene	1284.9	797.66	1.28	243.69±2.97[a]	123.29±8.22[b]	93.01±8.15[c]
V9	3-Methyl-1-butanol-M	1218.1	694.72	1.24	3153.66±226.35[a]	2891.66±100.97[a]	2938.37±57.98[a]
V10	3-Methyl-1-butanol-D	1218.7	695.52	1.48	25896.75±118.09[a]	24354.47±126.97[b]	24441.69±130.99[b]
V11	Ethyl hexanoate	1241.8	729.61	1.80	339.00±10.34[b]	854.46±41.48[a]	918.35±38.86[a]
V12	alpha-Terpinene	1193.8	660.64	1.22	95.78±2.19[a]	47.20±1.96[b]	37.10±2.76[c]
V13	1-Butanol-D	1157.5	586.91	1.39	1970.69±31.74[a]	1544.81±43.40[b]	1498.87±17.63[b]
V14	Isoamyl acetate	1135.1	544.89	1.74	11822.90±3.32[b]	15511.23±95.06[a]	15577.80±197.56[a]
V15	1-Butanol-M	1158.3	588.49	1.26	3747.20±21.65[a]	3493.41±49.02[b]	3407.02±19.45[c]
V16	2-Methyl-1-propanol-M	1105.6	494.15	1.26	8374.85±48.96[a]	7566.32±41.98[c]	7737.80±40.66[b]
V17	2-Methyl-1-propanol-D	1105.2	493.36	1.37	20085.28±17.28[a]	18822.61±151.27[b]	18675.70±120.55[b]
V18	Isobutyl propionate	1094.6	476.71	1.70	69.71±9.14[b]	333.78±2.28[b]	351.44±13.18[a]
V19	Butyl acetate	1079.7	456.15	1.64	78.93±4.60[c]	162.95±2.49[b]	152.04±7.81[a]
V20	Ethyl-3-methylbutanoate	1063.8	435.17	1.64	945.57±1.05[c]	1533.69±10.58[b]	1568.95±25.74[a]
V21	Ethyl butanoate	1049.6	417.18	1.55	5685.13±27.84[a]	5111.76±15.73[b]	5111.14±44.18[b]
V22	Isobutyl acetate	1026.2	389.20	1.61	3320.47±6.51[b]	4596.89±22.79[a]	4601.46±28.31[a]
V23	2-Butanol	1036.0	400.69	1.33	13808.30±51.24[b]	14036.12±76.64[a]	13971.64±75.55[a]
V24	Propyl acetate	991.4	352.22	1.46	2101.36±2.86[c]	2490.60±14.22[a]	2441.01±15.27[b]
V25	Ethyl isobutyrate	979.9	343.23	1.56	1497.35±7.63[b]	1873.64±5.17[a]	1870.65±8.37[a]
V26	Ethyl propanoate	971.4	336.73	1.45	8961.24±64.39[b]	9197.82±66.67[a]	9281.93±46.64[a]
V27	Ethanol	943.6	316.25	1.12	56329.32±59.57[a]	54593.18±21.92[b]	54317.23±26.04[b]
V28	Ethyl Acetate	899.4	286.27	1.33	20532.04±245.08[a]	20475.16±167.67[a]	19952.63±9.60[b]
V29	2-Butanone	915.3	296.76	1.24	2775.39±13.98[c]	3024.19±20.66[b]	2813.14±5.06[a]
V30	Methanol	887.6	278.78	0.97	518.52±28.34[a]	518.80±19.24[a]	551.02±3.22[a]
V31	Ethyl formate	864.7	264.79	1.05	354.04±87.03[a]	201.68±8.57[b]	48.74±2.33[c]
V32	Acrolein	858.8	261.29	0.97	680.87±28.95[c]	722.83±9.96[b]	881.42±7.55[a]
V33	Methyl acetate	849.4	255.79	1.19	2966.82±223.94[a]	3270.21±6.21[a]	3253.89±76.71[a]
V34	Acetone	838.9	249.80	1.11	3949.18±60.78[c]	3443.89±92.58[b]	3225.21±20.45[a]
V35	Propanal	819.8	239.30	1.14	3224.72±204.03[c]	2886.92±43.84[b]	2215.50±28.60[a]
V36	2-Methylpropanal	832.6	246.30	1.28	735.72±48.91[a]	663.67±38.21[ab]	635.17±18.54[b]
V37	Acetaldehyde	768.8	213.32	1.02	2427.39±113.46[a]	2110.36±36.23[b]	1883.92±45.27[c]
V38	1-Propanol	1054.0	422.67	1.25	4819.91±3.51[c]	5034.08±2.02[a]	4963.56±23.08[b]

V39	Propyl propanoate	1001.9	362.22	1.58	40.74±4.04[c]	112.05±1.65[b]	125.23±3.30[a]
V40	Ethyl decanoate	1780.7	2337.27	1.62	865.41±251.93[a]	1020.73±353.94[a]	1112.16±37.80[a]
V41	Methyl benzoate	1632.5	1694.51	1.20	258.14±42.69[b]	278.82±19.51[b]	350.68±26.26[a]
V42	1-Hexanol	1358.3	934.56	1.30	714.38±36.07[b]	918.97±22.91[a]	966.74±29.26[a]
V43	3-Methylbutyl propanoate-M	1194.8	661.91	1.35	310.15±1.54[c]	657.22±6.99[b]	714.02±9.96[a]
V44	3-Methylbutyl propanoate-D	1195.6	663.01	1.84	69.01±13.68[c]	283.41±11.48[b]	336.19±34.07[a]
V45	(Z)-3-Hexenol	1382.0	983.95	1.22	69.16±10.69[a]	57.01±6.62[a]	62.87±4.23[a]
V46	1-Pentanol	1262.2	761.17	1.25	119.33±14.65[ab]	95.64±2.69[b]	105.93±2.52[a]
V47	Dimethyl sulfide	797.1	227.38	0.95	208.09±6.52[a]	119.85±8.34[c]	150.55±1.62[b]
V48	3-Methylbutanal	928.5	305.68	1.40	79.52±1.22[a]	65.86±2.18[b]	58.63±1.51[c]
V49	gamma-Terpinene	1261.5	760.07	1.20	164.83±4.61[b]	181.53±6.07[a]	180.55±3.63[a]
V50	Ethyl pentanoate	1147.6	567.95	1.69	111.52±3.76[b]	177.84±5.15[a]	177.92±6.13[a]
V51	unknown	807.6	232.81	1.31	13.17±5.05[a]	12.90±4.29[a]	1289.64±17.92[b]
V52	unknown	1050.8	418.68	1.38	1438.79±4.64[a]	1409.56±9.27[a]	1447.64±11.98[b]
V53	unknown	863.2	263.87	1.31	53.21±2.98[c]	102.03±6.00[b]	228.64±14.49[a]

Results are expressed as average (n = 3) ± standard deviation.

a, b, c—values followed by the same letter within a column do not differ significantly according to Tukey's test ($p < 0.05$).

RI: Represents the retention time; RT: Represents the retention index; DT: Represents the drift time; unknown: Not detected in sample. Y1: newly produced potato wine; Y2: ultrasonic treated potato wine; Y3: Gamma irradiated potato wine.
