# Supplementary material for: The Effects of Ultrasonic and Gamma Irradiation on the Flavor of Potato Wines Investigated by Sensory Omics
Source: Foods. 2023 Jul 25;12(15):2821. doi: 10.3390/foods12152821 (PMC10417215; doi:10.3390/foods12152821)
Supplement: Supplementary file 1 [file foods-12-02821-s001.zip › Table S5.pdf]

Table S5. The ROAV values of the main volatile compounds by GC-IMS.

Count	Compound	Thresholds (mg/kg)	Y1		Y2		Y3		Odor
			percentage (%)	ROAV	percentage (%)	ROAV	percentage (%)	ROAV	
V1	Propanoic acid	100	0.11±0.02^{c}	0.01	0.15±0.02^{b}	0.01	0.19±0.02^{a}	0.01	sour
V3	Acetic acid	120	0.40±0.03^{c}	0.03	0.48±0.05^{b}	0.03	0.57±0.01^{a}	0.02	acetic
**Total Acids**			0.51		0.63		0.76		
V2	Methional	40	0.31±0.03^{a}	0.07	0.17±0.02^{b}	0.03	0.13±0.00^{b}	0.02	meat
V32	Acrolein	0.21	0.31±0.02^{b}	13.63	0.33±0.01^{b}	11.13	0.40±0.01^{a}	13.33	irritant
V35	Propanal	0.2	1.48±0.09^{a}	68.30	1.31±0.02^{b}	46.37	1.02±0.02^{c}	35.70	grass
V36	2-Methyl-propanal	0.1	0.34±0.02^{a}	31.38	0.30±0.02^{b}	21.24	0.29±0.01^{b}	20.30	banana, muskmelon
V37	Acetaldehyde	100	1.11±0.02^{a}	0.10	0.96±0.02^{b}	0.07	0.86±0.01^{c}	0.06	grass
V48	3-Methylbutanal	0.2	0.04±0.00	1.85	0.03±0.00	1.06	0.03±0.00	1.05	nut
V29	2-Butanone	3	1.27±0.01^{b}	3.91	1.38±0.01^{b}	3.26	1.29±0.00^{a}	3.01	bitter
V34	Acetone	100	1.81±0.03^{a}	0.17	1.57±0.04^{b}	0.11	1.48±0.01^{c}	0.10	sweet, fruity
**Total Aldehydes, Ketones**			6.67		6.05		5.5		
V6	cis-2-Penten-1-ol	0.35	0.10±0.00^{a}	2.90	0.09±0.01^{a}	1.82	0.08±0.01^{b}	1.60	bitter, grass
V9	3-Methyl-1-butanol-M	0.7	1.45±0.10	19.12	1.32±0.06	13.35	1.35±0.03	13.50	bitter, almond
V10	3-Methyl-1-butanol-D	0.7	11.87±0.06	156.51	11.09±0.02	112.17	11.2±0.05	112.00	bitter, almond
V13	1-Butanol-D	10	0.90±0.02^{a}	0.83	0.70±0.02^{b}	0.5	0.69±0.01^{b}	0.48	wine, bitter
V15	1-Butanol-M	10	1.72±0.01^{a}	1.59	1.59±0.02^{b}	1.13	1.56±0.01^{c}	1.09	wine, bitter
V16	2-Methyl-1-propanol-M	8	3.84±0.03^{a}	4.43	3.44±0.01^{c}	3.04	3.55±0.02^{b}	3.10	oil
V17	2-Methyl-1-propanol-D	8	9.21±0.02	10.63	8.57±0.03	7.58	8.56±0.02	7.49	oil
V29	2-Butanol	5.1	6.33±0.03^{b}	11.46	6.39±0.02^{ab}	8.87	6.4±0.04^{a}	8.78	bitter
V27	Ethanol	2900	25.82±0.15^{a}	0.08	24.85±0.07^{b}	0.06	24.89±0.01^{b}	0.06	sweet, bitter
V30	Methanol	30	0.24±0.02^{a}	0.07	0.24±0.01^{a}	0.06	0.25±0.00^{a}	0.06	sweet, bitter
V38	1-Propanol	53.95	2.21±0.00^{c}	0.38	2.29±0.02^{a}	0.30	2.27±0.01^{b}	0.29	flower
V42	1-Hexanol	0.2	0.33±0.02^{b}	15.23	0.42±0.01^{a}	14.87	0.44±0.02^{a}	15.4	fruity
V45	(Z)-3-Hexenol	1	0.03±0.01^{a}	0.28	0.03±0.01^{a}	0.21	0.03±0.00^{a}	0.21	greenery
V46	1-Pentanol	5	0.05±0.01^{a}	0.09	0.04±0.00^{b}	0.06	0.05±0.00^{a}	0.07	bitter
**Total Alcohols**			64.1		61.05		61.32		
V4	Ethyl octanoate	0.02	0.30±0.01^{c}	138.45	0.46±0.01^{b}	162.84	0.50±0.01^{a}	175	brandy, fruity
V5	Ethyl lactate	128	0.42±0.03^{b}	0.03	0.53±0.05^{a}	0.03	0.53±0.05^{a}	0.03	oil, bitter
V11	Ethyl hexanoate	0.21	0.16±0.01^{b}	7.03	0.39±0.02^{a}	13.15	0.42±0.02^{a}	14	fruity
V22	Isoamyl acetate	0.5	5.42±0.01^{b}	100	7.06±0.01^{a}	100	7.14±0.09^{a}	100	pear
V18	Isobutyl propionate	0.02	0.03±0.01^{b}	13.85	0.15±0.02^{a}	53.10	0.16±0.01^{a}	56.00	fruity
V19	Butyl acetate	0.5	0.04±0.01^{b}	0.74	0.07±0.01^{a}	0.99	0.07±0.00^{a}	0.98	fruity
V20	Ethyl-3-methylbutanoate	0.2	0.43±0.00^{c}	19.84	0.70±0.00^{b}	24.78	0.72±0.01^{a}	25.2	sweet, apple

V21	Ethyl butanoate	0.08	2.61±0.01^{a}	301.13	2.33±0.02^{b}	206.21	2.34±0.02^{b}	204.75	pineapple, oil
V14	Isobutyl acetate	0.3	1.52±0.00^{b}	46.77	2.09±0.01^{a}	49.32	2.11±0.02^{a}	49.93	fruity
V24	Propyl acetate	0.8	0.96±0.00^{b}	11.08	1.13±0.02^{a}	10.00	1.12±0.01^{a}	9.80	strawberry, pear
V25	Ethyl isobutyrate	0.057	0.69±0.01^{b}	111.73	0.85±0.01^{a}	105.58	0.86±0.01^{a}	105.61	osmanthus, fruity
V26	Ethyl propanoate	19.19	4.11±0.03^{b}	1.98	4.19±0.04^{a}	1.55	4.25±0.02^{a}	1.55	banana, fruity
V28	Ethyl Acetate	32.55	9.41±0.13^{b}	2.67	9.32±0.07^{b}	2.03	9.14±0.01^{a}	1.97	pineapple
V31	Ethyl formate	6.6	0.16±0.04^{a}	0.22	0.09±0.01^{b}	0.10	0.02±0.00^{c}	0.02	peach, irritant
V33	Methyl acetate	1.5	1.36±0.10^{a}	8.37	1.49±0.08^{a}	7.03	1.49±0.00^{a}	6.95	fruity
V39	Propyl propanoate	57	0.02±0.00	0.003	0.05±0.00	0.006	0.06±0.00	0.007	alcohol
V40	Ethyl decanoate	1.12	0.40±0.12^{a}	3.30	0.46±0.16^{a}	2.91	0.51±0.07^{a}	3.19	rose, fat
V41	Methyl benzoate	1.433	0.12±0.02^{b}	0.77	0.13±0.01^{b}	0.64	0.16±0.01^{a}	0.78	flower
V43	3-Methylbutyl propanoate-M	ND	0.14±0.00^{c}	ND	0.30±0.00^{b}	ND	0.33±0.01^{a}	ND	plum, almond
V44	3-Methylbutyl propanoate-D	ND	0.03±0.01^{c}	ND	0.13±0.00^{b}	ND	0.15±0.02^{a}	ND	plum, almond
V50	Ethyl pentanoate	0.094	0.05±0.01	4.91	0.08±0.01	7.86	0.08±0.02	5.96	fat
	**Total Esters**		28.38		31.3		31.44		
V7	Terpinolene	0.875	0.04±0.01^{a}	0.42	0.03±0.01^{a}	0.24	0.04±0.00^{a}	0.32	orange
V49	gamma-Terpinene	1	0.08±0.01^{a}	0.74	0.08±0.01^{a}	0.57	0.08±0.00^{a}	0.56	leaves
V12	alpha-Terpinene	0.08	0.04±0.01	4.62	0.02±0.00	1.77	0.02±0.02	3.75	lemon
V8	p-Cymene	0.01	0.11±0.00^{a}	101.53	0.06±0.01^{b}	42.48	0.04±0.01^{c}	28	wood
V47	Dimethyl sulfide	ND	0.10±0.01^{a}	ND	0.06±0.01^{c}	ND	0.07±0.00^{b}	ND	vegetable
	**Total Others**		0.37		0.25		0.25		

Results are expressed as average (*n* = 3) ± standard deviation.

a, b, c—values followed by the same letter within a column do not differ significantly according to Tukey's test ($p < 0.05$).

Y1: newly produced potato wine; Y2: ultrasonic treated potato wine; Y3: Gamma irradiated potato wine; ND: Not detected in sample.
